# How Digital Are the Digital Humanities? An Analysis of Two Scholarly Blogging Platforms

**DOI:** 10.1371/journal.pone.0115035

**Published:** 2015-02-12

**Authors:** Cornelius Puschmann, Marco Bastos

**Affiliations:** 1 Faculty of Social Sciences, Zeppelin University, Am Seemooser Horn 20D, Friedrichshafen D-88045, Germany; 2 Franklin Humanities Institute, Duke University, 114 S. Buchanan Blvd, Bay 5 Box 90403, Durham, North Carolina 27701, United States of America; Université de Montréal, CANADA

## Abstract

In this paper we compare two academic networking platforms, HASTAC and Hypotheses, to show the distinct ways in which they serve specific communities in the Digital Humanities (DH) in different national and disciplinary contexts. After providing background information on both platforms, we apply co-word analysis and topic modeling to show thematic similarities and differences between the two sites, focusing particularly on how they frame DH as a new paradigm in humanities research. We encounter a much higher ratio of posts using humanities-related terms compared to their digital counterparts, suggesting a one-way dependency of digital humanities-related terms on the corresponding unprefixed labels. The results also show that the terms digital archive, digital literacy, and digital pedagogy are relatively independent from the respective unprefixed terms, and that digital publishing, digital libraries, and digital media show considerable cross-pollination between the specialization and the general noun. The topic modeling reproduces these findings and reveals further differences between the two platforms. Our findings also indicate local differences in how the emerging field of DH is conceptualized and show dynamic topical shifts inside these respective contexts.

## Introduction

The advent of the Internet has profoundly affected scholarly communication [1–4]. Few scholars, whether in the sciences, social sciences, or humanities can imagine conducting research or organizing teaching without relying on email, digital library services, or e-learning environments. Formal academic publishing has undergone a series of changes with the increased availability of electronic publications, whether under an open access or toll access regime [5]. Structural changes in the dissemination of knowledge have largely been gradual and evolutionary: while the volume of scholarly publications has greatly increased in the past decades and the formal and distribution models have diversified, the form and function of research articles and scholarly monographs have remained relatively stable [6].

Meanwhile, the range of avenues available for the dissemination of informal scholarly communication has increased exponentially. In addition to formal publication venues, scholars can now communicate their findings in (micro)blogs, wikis, social networking sites (SNS) and countless other social web platforms [7–10]. Such services carry both opportunities and risks for early-career researchers, and they are used for a wide variety of purposes and with a range of motives [11–13]. While researchers are able to disseminate their findings more quickly and reach out to broader audiences than was previously possible, they also risk that their work will not be acknowledged in more traditional and hierarchical professional structures. Informal genres of scholarly communication frequently lack peer review and rely on new measures of impact, rather than the established currency of acceptance within a field [14]. As a result, researchers have overall been very careful in their acceptance of digital formats that compete with established forms of expert knowledge dissemination, largely choosing instead to focus on established formats [15]. This is especially true in the humanities, where conservatism towards new formats is particularly strong.

Digital Humanities (DH) can be broadly characterized as the adoption of an array of computational methodologies for humanities research [16, 17]. During the early nineties, DH scholarship developed under the umbrella of several academic organizations dedicated to what was then commonly referred to as humanities computing [18]. These organizations brought together scholars from different fields interested in exploring computational methods for traditionally-defined humanities scholarship [19]. The suffix “digital” is increasingly used to delineate the new computational areas of humanities research (i.e. digital literature, digital archaeology, digital history, etc.). The introduction of computational methods aims among other things to supplement established humanities research routines and explore new methodological avenues, such as text analysis and encoding; archive creation and curation; mapping and GIS; and modeling of archaeological and historical data [20, 21].

Since the early 2000s the term Digital Humanities has also been used to refer to humanities research defined by a data-driven approach, in which summarization and visualization are important methodological cornerstones. Media and cultural studies, library and archival studies, digital pedagogy, and the recently emergence of MOOCs have also been referred to as Digital Humanities in a more general sense [22]. As a result, DH has evolved to incorporate a range of different definitions and is subject to considerable interpretative flexibility [23]. The central hypothesis of this study is that the variety of terms and topics associated with DH is locally configured, and that their makeup reflects different (and to a degree contradictory) conceptualizations of what constitutes DH.

## DH and Social Media

Because of its interdisciplinary and international character, its affinity for digital media, and its recent emergence as a scholarly movement, DH has been comparatively strongly impacted by informal communication tools such as blogs and Twitter, with junior scholars invested in DH research using such tools widely to organize, network, and collaborate. Kirschenbaum notes the important role of social media for establishing and galvanizing DH as a movement: “Twitter, along with blogs and other online outlets, has inscribed the digital humanities as a network topology, that is to say, lines drawn by aggregates of affinities, formally and functionally manifest in who follows whom, who friends whom, who tweets whom, and who links to what.” [17] Usage of Twitter and blogs has contributed to establishing DH as a brand, and it has helped to increase its visibility on a global scale [24]. While actively using social media does not make one a digital humanist, social media applications seem to be perceived as valuable instruments for intra-community communication in the DH community, rather than being used just out of curiosity or for self-promotion [11]. Crucially, there are scholars who take up blogging and Twitter because they are important channels of communication in the DH community.

Such tools therefore increasingly constitute scholarly infrastructure to their users in the same sense that library services and communal mailing lists constitute infrastructure. While traditional scholarly organizations are struggling to integrate social media, DH scholars, especially junior researchers, have considerable uptake of such tools, reflected for example in the strong use of Twitter at the annual Digital Humanities conference [24, 25]. DH can therefore be characterized as an *emerging digital scholarly network*—a group of scholars that has integrated digital genres of scholarly communication into its communicative infrastructure from the onset. Inside such a network in which heterogeneous links connect different actors it should be possible to study the flow of ideas, trends, and discourses much more effectively through social media than purely by assessing formal publications in scholarly journals and monographs [26].

### HASTAC

The Humanities, Arts, Science, and Technology Alliance and Collaboratory (HASTAC) is an online community and social network that connects researchers, young scholars, and the general public interested in a wide range of subjects associated with DH and peer-to-peer learning. Founded in 2002 by Davidson and Goldberg [27], HASTAC emerged as a consortium of educators, scientists, and technology designers funded by the National Science Foundation, the Digital Promise Initiative, and the MacArthur Foundation, with infrastructure provided by Duke University and the University of California Humanities Research Institute. HASTAC differs from similar initiatives in that it is largely decentralized with content generated by a network of over ten thousand members including university faculty, students, and general public.

The network platform is built on the Drupal content management system and requires an inclusive free-of-charge membership. Member participation varies widely, with many registering but passively interacting with the website by reading the content and a robust minority expressing their thoughts and communicating their interests by writing or commenting on blog posts, joining discussion forums, or contributing information about current events. According to the initiative’s website, “HASTAC members are motivated by the conviction that the digital era provides rich opportunities for informal and formal learning and for collaborative, networked research that extends across traditional disciplines, across the boundaries of the academy and the community, across the two cultures of humanism and technology, across the divide of thinking versus making, and across social strata and national borders.” [28]. While the platform is interdisciplinary in nature, it is strongly focused on learning and DH-related topics.

### Hypotheses

Hypotheses is a publication platform for academic blogs. Launched in 2004, it is funded and operated by the Centre for Open Electronic Publishing (Cléo), a unit that brings together two major French research institutions and two universities: the Centre national de la recherche scientifique (CNRS), the École des Hautes Études en Sciences Sociales (EHESS), the Aix-Marseille Université, and the Université d’Avignon. In addition to Hypotheses, Cléo provides other tools via the OpenEdition portal: Revues.org, a platform for journals in the humanities and social sciences and Calenda, a calendaring tool.

According to the Hypotheses website “[a]cademic blogs can take numerous forms: accounts of archaeological excavations, current collective research or fieldwork; thematic research; books or periodicals reviews; newsletter etc. Hypotheses offers academic blogs the enhanced visibility of its humanities and social sciences platform. The Hypotheses team provides support and assistance to researchers for the technical and the editorial aspects of their project.” [29] To publish on Hypotheses, a blog must first be admitted by the platform’s editorial team. Only researchers employed by institutions of higher learning are eligible to join Hypotheses after having been evaluated, and the criterion for positive evaluation is a consistent focus on academic issues. Through its policy the platform maintains some characteristics of a formal publication outlet, aiming to stimulate both open discussion within scholarly disciplines and exchange with the broader public.

Hypotheses is based on the Wordpress content management platform, with a home page that features current contributions from participant blogs. In addition to English, a large portion of Hypotheses’ content is composed in French, German, Spanish, and other languages, but for the purpose of this study we only considered posts published in English.

### Similarities and differences

Both platforms share strong similarities: they aim to promote new forms of scholarly communication and knowledge dissemination. At the same time, there are also considerable differences: HASTAC places a clear emphasis on learning and also mentions media and communication in its self-characterization. While Hypotheses is also interdisciplinary in character, it has a stronger slant towards traditional humanities subfields, and specifically towards history. The concept of scholarly blogging outlined on the Hypotheses website points to its role for intradisciplinary communication, whereas HASTAC is more geared towards interdisciplinary exchange. Despite these differences, the two platforms make an ideal case for comparison on the grounds of their functional similarities. Both are related to DH, both seek to integrate blogging into scholarly communication, and both are publicly funded. Furthermore, both platforms have been operational for a similar timespan and attract broadly comparable user communities.

## Research Design

Our aim is to characterize differences in the discourse that takes place on HASTAC and Hypotheses reflecting different cultural implementations of DH and different understandings of what constitutes DH. To this end, we formulated two research questions: How frequent are particular keywords associated with (digital) humanities on the two platforms (ᴴ1) and what are thematic differences in the distribution of topics in the two sites (ᴴ2)? We approached the first question by counting the co-occurrence of humanities-related terms and their digital equivalents (e.g. history—digital history) on blog posts. In a second step we applied topic modeling to the post content to identify substantial thematic differences between the communities in both platforms and their respective approaches to blogging. Based on the self-characterizations of both platforms, we expected there to be both overlap and variation with regards to the adoption of DH-related labels and overall disciplinary focus.

### Data

The data from the two platforms were collected from database dumps containing the SQL table structure and the blog post content. HASTAC data included content posted between August 14, 2006 and August 14, 2013, together with the profile data of 11,284 users. Most users shared brief biographical information and identified a set of topical interests, institutional affiliation, and links to personal websites. In addition to the posts themselves, the Hypotheses data included metadata such as author information, timestamp, text, internal and external links in each post, which was collected between the 1st of July 2006 and the 30rd of June 2012.

**Fig 1:**
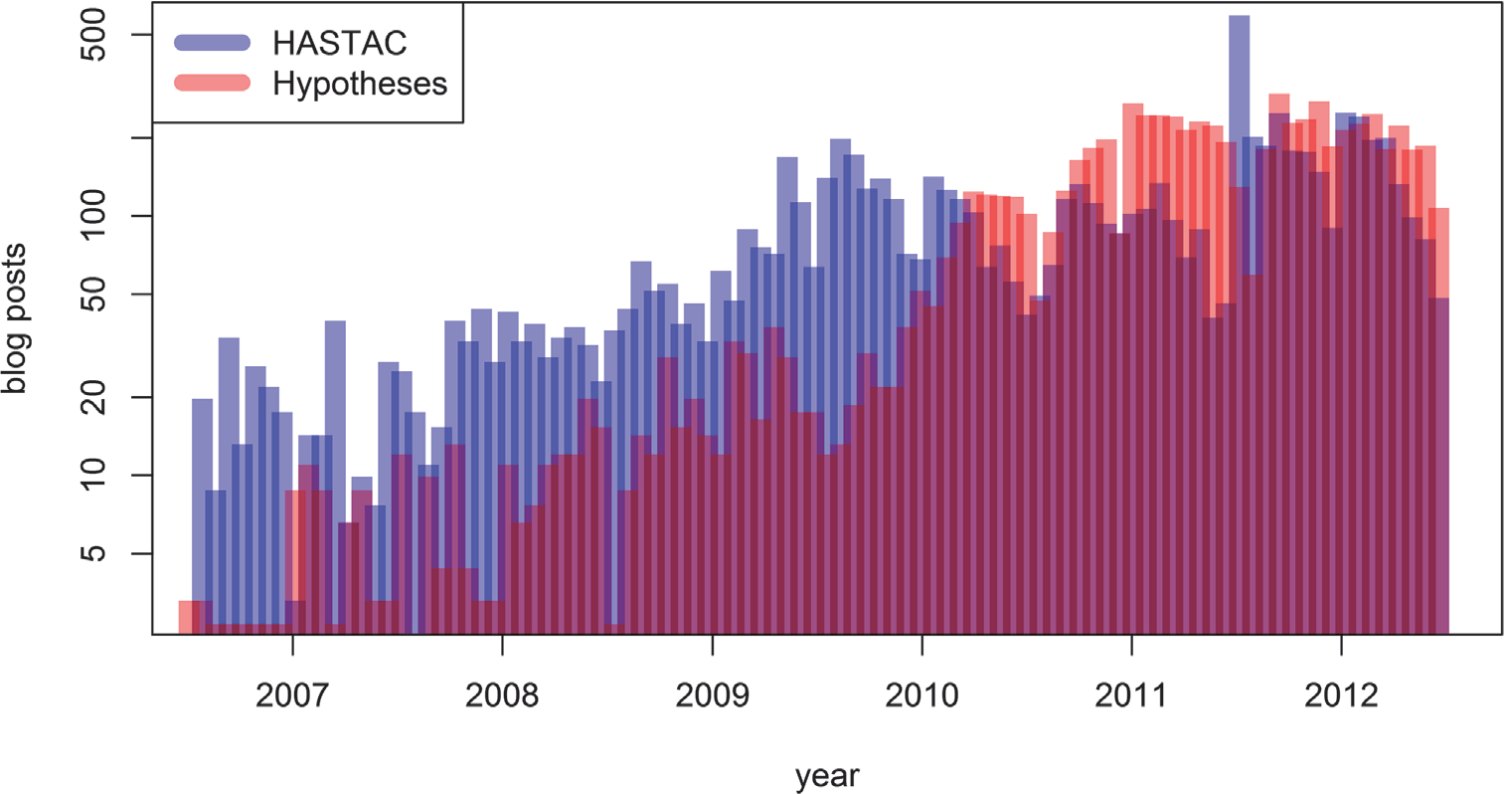
English-language blog posts published on both sites between 2006 and 2012.

The language of posts was detected automatically using the language identification system *langid*.*py* for Python, which supports a large number of languages and achieves a high level of accuracy without requiring prior in-domain classifier training [30]. The material initially included a large number of posts published in languages other than English (45,528 posts) published over different periods of time. For the purpose of this investigation, we only considered blog posts in English published between the 1^st^ of July 2006 and the 30^th^ of June 2012, thus extracting 7,269 posts from HASTAC and 6,777 posts from Hypotheses. We performed a co-word analysis over these 14,046 posts [31] and subsequently extracted a random sample of 5,000 posts from each platform to perform topic modeling. Fig. 1 shows a frequency histogram of blog posts in the abovementioned period on a logarithmic scale, with HASTAC posts being comparatively more frequent from 2006 to 2010, and posts on Hypotheses being comparatively more frequent in the period thereafter. Activity on both platforms drops during the summer vacation months (July for HASTAC and August for Hypotheses) reflecting seasonal work patterns.

### Methods

We approached our first question (ᴴ1) by means of a co-word analysis of keywords associated with humanities and Digital Humanities research [31]. We used one vector of twenty humanities areas (anthropology, archaeology, archive, art, culture, ethnography, history, humanities, learning, libraries, literacy, literature, media, pedagogy, preservation, publishing, rhetoric, scholarship, storytelling, knowledge) and another identical vector plus the suffix “digital” (digital anthropology, digital archaeology, digital archive, digital art, digital culture, digital ethnography, digital history, digital humanities, digital learning, digital libraries, digital literacy, digital literature, digital media, digital pedagogy, digital preservation, digital publishing, digital rhetoric, digital scholarship, digital storytelling, digital knowledge). These keywords include terms that describe fields or general domains associated with the humanities on the basis of raw token frequencies identified in the two datasets. This approach comes with considerable limitations. Firstly, the semantics of the terms differ considerably, as some describe fields of scholarship (history—digital history), while others are more general and tend to be polysemous (knowledge, media). The same applies to their prefixed counterparts, with digital history likely identifying a field, while digital media most likely describes certain kinds of technical media. Furthermore, issues of precision and recall arise, due to which not all discussion of the relevant phenomena is reliably captured and some of what is captured relates to other concepts. In spite of these limitations, we found co-word analysis to be useful, because it shows the entrenchment of the terms as convenient and fashionable labels on both platforms. We accept that such labels do not narrowly identify concepts, but believe that they are suitable to characterize the success of particular terms around which the DH community can rally.

Using these terms we generated a series of term-document matrices for each of the networks. We visualized the association between humanities and DH by performing a multinomial logistic regression on the terms. We relied on the *textir* package for R [32] to convert the term-to-term co-occurrence matrix to a matrix of the log-odds ratios of co-occurrence. The resulting matrices (HASTAC and Hypotheses) scales the word similarity as a function of word frequency, with terms of similar semantic content numerically represented as being similar to one another [33]. After converting the log-odds ratios to distance matrices using cosine similarity [34, 35], we relied on multidimensional scaling [36] to visualize humanities and DH terms in a latent semantic space [37] with a two-dimensional density surface [38].

The second question (ᴴ2) was addressed using Latent Dirichlet Allocation [39] implementation for R [40]. R package *topicmodels* allows the probabilistic modeling of term frequency occurrences in documents and estimation of similarities between documents and words using an additional layer of latent variables referred to as topics. The package provides the basic functions for fitting topic models based on data structures from the text mining package *tm* [41]. Topics were modeled using a mixed-membership approach in which documents are not assumed to belong to single topics, but to simultaneously belong to several topics, with varying distributions across documents. To equally represent both platforms, we drew a random sample of 5,000 posts from each platform from the data previously described. Prior to mapping the documents to the term frequency vector, we tokenized the posts and processed the tokens by removing punctuation, numbers, stemming, and stop words, in order to sparsen the matrices. We also omitted very short documents (<200 characters) for the same purpose.

**Ethics Statement.** The authors confirm that the study is in compliance with the Terms and Conditions of HASTAC and Hypotheses.

## Results

### Co-word analysis

With respect to our first research question (ᴴ1) we found that unprefixed keywords occurred in a much higher ratio relative to their prefixed counterparts. Table 1 shows the number of occurrences of humanities and DH terms on both platforms, with a high concentration of posts focusing on art, media, history, culture, and humanities, followed by learning, publishing, and libraries. The areas of research with fewer occurrences are archaeology, storytelling, ethnography, and preservation. HASTAC presented a much higher number of references to humanities (21,262) and DH (2,771) in comparison to Hypotheses (9,644 and 187, respectively). The ratio of posts with humanities to DH related terms is also higher on HASTAC at seven posts on humanities to each post on DH while on Hypotheses the ratio is of fifty-one posts on humanities to each post on DH. In fact, we found no mention to nine areas of DH in the Hypotheses sample.

**Table 1:** Number of occurrences of humanities and DH terms.

	HASTAC HU	HASTAC DH	Hypo HU	Hypo DH
anthropology	213	4	310	NA
archaeology	80	2	147	NA
archive	784	99	365	11
art	3363	128	1626	8
culture	1540	146	1168	12
ethnography	144	8	117	NA
history	1772	38	1945	15
humanities	1533	674	309	69
knowledge	1515	15	613	NA
learning	2026	113	187	NA
libraries	924	116	293	34
literacy	505	92	45	NA
literature	727	10	492	NA
media	3118	1021	761	17
pedagogy	504	27	48	NA
preservation	139	31	90	7
publishing	1150	35	843	11
rhetoric	309	11	76	NA
scholarship	735	132	190	1
storytelling	181	69	19	2

**Fig 2:**
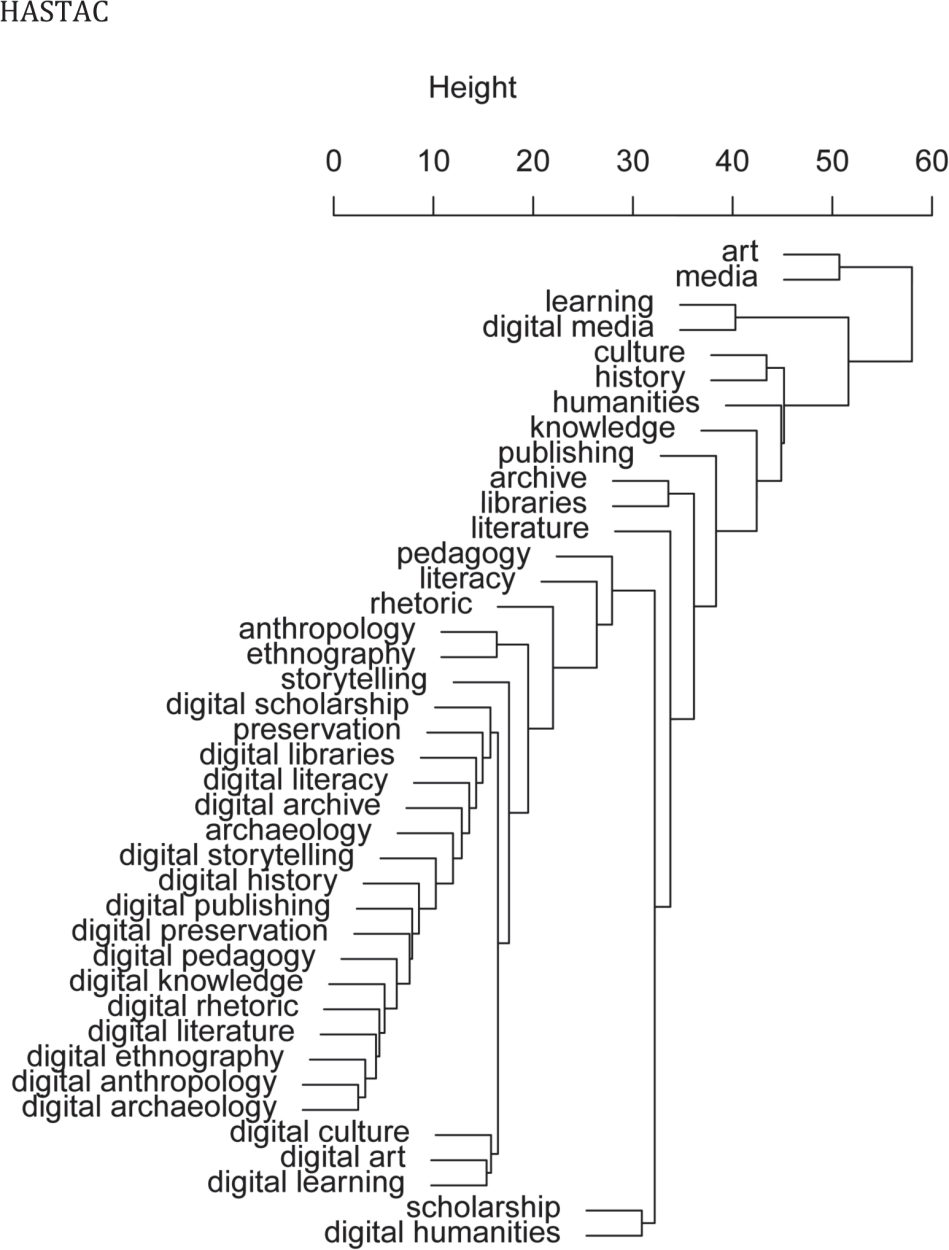
Hierarchical cluster dendrogram of term co-occurrences in both platforms.

Although the distribution of humanities and DH terms is skewed towards HASTAC, the distribution per area of research on humanities is fairly similar. Fig. 2 shows a cluster dendogram of term co-occurrences based on Euclidean distance, with humanities areas appearing at the top of the hierarchical structure and DH terms appearing near the bottom. Art, culture, and media are likely to also refer to general terms rather than only humanities disciplines, therefore presenting a higher value of intergroup dissimilarity and appearing higher up in the hierarchy. More narrowly defined areas such as learning and digital media are followed on HASTAC, while the hierarchical clustering of topics on Hypotheses is topped by history and publishing. Fig. 2 shows internal differences and dissimilarities between the two platforms in their usage of the labels listed in Table 1.

DH subfields are much more distinct from other terms in HASTAC that they are on Hypotheses, where many of the DH labels are either uncommon or not used at all. Unsurprisingly, we found that most blog posts that made reference to DH terms also included references to the unprefixed terms, but not the other way around. From the 5,711 posts on HASTAC that included references to humanities-related terms (21,262 occurrences), 89% of them also included references to the corresponding label in DH. However, from the 1,996 posts on HASTAC that included references to Digital Humanities terms (2,771 occurrences), only 11% of them also included references to the corresponding term in the humanities. This asymmetry is actually more pronounced in the Hypotheses network. From the 4,001 posts on Hypotheses that included references to humanities-related terms (9,644 occurrences), 98% also included references to the corresponding term in DH. However, from the 140 posts on Hypotheses that included references to DH-related terms (187 occurrences), only 2% also included references to the corresponding humanities area.

The dependence of Digital Humanities on established humanities labels is consistent, but it varies considerably within each of the areas investigated. The average percentage of posts per area that include reference to both humanities and DH is still quite skewed, as 80% of posts on HASTAC (mean = .79, median = .84) and Hypotheses (mean = .78, median = .81) dedicated to Digital Humanities areas also including references to the main humanities area. The reverse dependency is also observed in the aggregated data per area, as less than 10% of posts on HASTAC (mean = .09, median = .05) and Hypotheses (mean = .05, median = .02) dedicated to humanities also included references to the related DH area. However, the dependency is noticeably lower in some fields of humanities. Preservation and archival studies presented a much lower ratio of posts dedicated to Digital Humanities that also referred to the associated humanities area (48% and 74% on HASTAC, and 57% and 91% on Hypotheses). Storytelling, literacy, and pedagogy are also particularly independent in the HASTAC network, with 52%, 63%, and 67% of posts making reference to digital terminology without mentioning the related humanities field. On Hypotheses, art is the term most detached from the main humanities area, with 63% of posts dedicated to digital art not making reference to the unprefixed field.

Some areas show a strong intersection of humanities and DH terms. A considerable proportion of articles that refer to humanities, storytelling, and libraries also made reference to digital humanities, digital storytelling, and digital libraries (37%, 20%, and 11% on HASTAC, and 21%, 11%, and 9% on Hypotheses). Media, scholarship, literacy, and preservation also presented higher-than-average levels of cross-pollination on HASTAC, with 30%, 14%, 11%, and 11% of the articles focusing on these terms also making reference to their niche Digital Humanities label. Most of these terms also presented a considerable level of intersection of DH with general terms.

**Fig 3:**
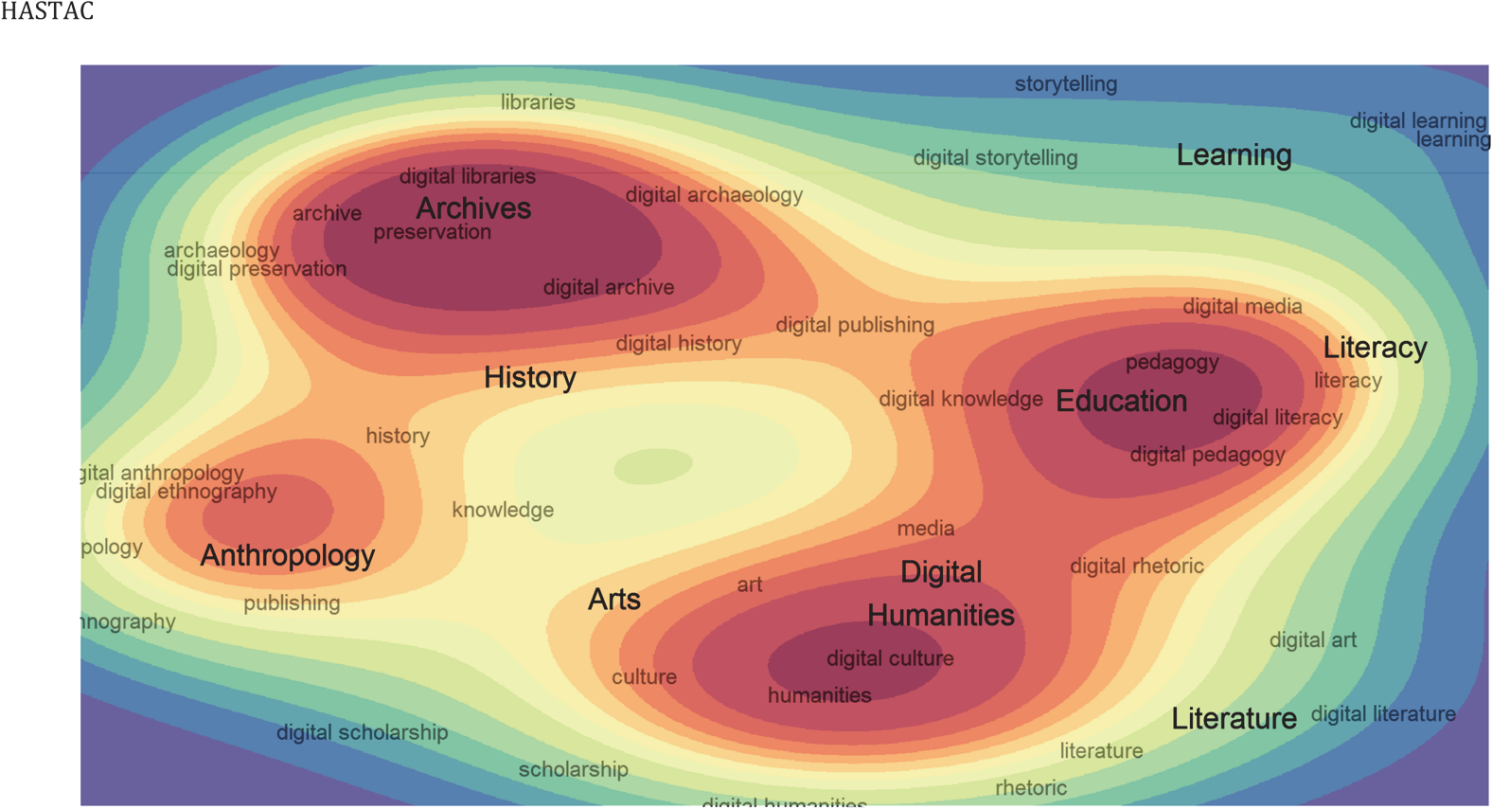
Density curves of log-odds co-occurrence ratios between humanities-related terms. Larger labels represent thematic areas manually identified.

We further explored the interplay between humanities and DH by performing a multinomial logistic regression on the terms. The matrices of log-odds ratios of co-occurrence indicate the word similarity and allow for visualizing humanities and DH terms in a latent semantic space with a two-dimensional density surface. Fig. 3 shows a contour-sociogram of the terms with substantial cross-pollination across different topics of humanities and Digital Humanities research. HASTAC posts with humanities and DH terms are clearly clustered around four main groups. The first includes terms associated with humanities at large, culture, and arts; the second is dedicated to education and learning; the third to archives and libraries; and the last clusters terms associated with anthropology and history. On the other hand, Hypotheses posts with humanities and DH terms are mostly concentrated on a single cluster due to many topics lacking more entry points. Nonetheless, humanities content published on Hypotheses presents clusters around humanities and media; archives, history, and arts; and one cluster grouping library-related materials.

The vast majority of articles focusing on digital media, digital libraries, digital art, digital humanities, digital culture, and digital publishing also included references to the main humanities area. This is particularly the case on HASTAC (93%, 91%, 89%, 85%, 84%, and 80%, respectively), but also on Hypotheses (71%, 79%, 63%, 96%, 83%, and 91%, respectively). In short, the results predictably show a considerable one-way dependency of DH on the unprefixed keyword, and a relative independence of the latter relative to the former. However, there are a few DH areas that presented substantial independence from the related humanities area, namely preservation, archive, storytelling, literacy, and pedagogy. We interpret this emancipation as an indicator for the establishment of these terms as convenient labels, which, while not necessarily identifying clear-cut concepts, provide attractive brands for the DH community to rally around.

### Topic modeling

We proceeded by exploring the topical differences between the two platforms to test our second research question (ᴴ2). We modeled twenty topics for the combined corpus of both platforms (5,000 posts each). Table 2 provides an overview of twelve selected topics and their ten most distinct terms by rank, some of which related to particular domains (Health, History, Law, Art, Games), while others are related to more general themes (Chatter, Learning). Topics were labeled through a qualitative interpretation of the most salient topic keywords and reading a sample of the associated blog posts, meaning that they retain a certain subjective bias. Most domain areas identified are strongly associated with content published on Hypotheses through individual blogs with a clear and consistent topical focus (e.g. Health, History, Law, Energy), while HASTAC has a stronger association with metatopics such as Learning, Data, and Gaming.

**Table 2:** Common topics on HASTAC and Hypotheses.

**Topic 1: Health**	**Topic 2: Cold War**	**Topic 3: Law**	**Topic 4: DH**
health	war	law	digital
medicine	university	legal	humanities
medical	korean	series	university
history	history	turkish	hastac
food	korea	history	new
university	cold	also	media
social	culture	said	will
urban	women	one	scholars
care	art	book	technology
research	visual	new	research
**Topic 5: SocMed**	**Topic 6: Data**	**Topic 7: Art**	**Topic 8: Urban Std**
social	can	university	urban
can	data	art	social
new	use	museum	political
media	will	history	new
one	digital	heritage	international
cultural	information	museums	studies
culture	project	cultural	european
time	also	music	global
digital	site	new	economic
space	work	sound	management
**Topic 9: Gaming**	**Topic 10: Chatter**	**Topic 11: Learn**	**Topic 12: Energy**
game	one	students	energy
games	people	learning	climate
video	like	will	change
play	can	can	policy
virtual	just	class	countries
world	time	new	will
one	even	education	global
can	think	digital	gas
gaming	now	one	carbon
worlds	many	work	paper

Some topics of general interest (e.g. Social Media and Data) are shared between the platforms. Conference Calls and Job Advertisements form two distinct yet evenly distributed topic based on their stylistic uniformity. In addition to pointing out thematic differences, topics also reflect differences in style between the two sites. Topic #12 (Chatter) is lexically distinct from other topics in that it uses much more general nouns (*time*, *people*) and verbs (*think*, *know*). It reflects a set of essayistic posts, particularly on HASTAC, which discuss controversial issues and tend to be relatively short. Spam is also a distinct topic, but one that is also shared between both sites.

We also found that while some topics overlap somewhat, many are highly characteristic of one of the two platforms. Topics #1 (Health), #2 (Cold War), #4 (Law), #8 (Art), #9 (Urban Studies), and #16 (Energy) are relatively clearly associated with Hypotheses, while topics #5 (Digital Humanities), #10 (Gaming), #12 (Chatter), and #15 (Learning) are prevalent on HASTAC. Topics #6 (Social Media) and #7 (Data) show a more even distribution between the two sites. Similar to our findings in the co-word analysis, #5 (Digital Humanities) is more prevalent in HASTAC than in Hypotheses. The distribution of topic scores suggests that a number of linguistically distinct thematic areas exist on Hypotheses, and that these areas follow disciplinary patterns. By contrast, HASTAC posts are less clearly associated with a single field of inquiry and most closely associated with metatopics such as learning and general conversation. HASTAC posts are also linked to the discussion of Digital Humanities and the usage of labels related to DH. The differences between the two platforms may point to diverging goals associated with scholarly blogging: addressing broad interdisciplinary issues before a wider public vs. conducting focused scholarly discussion within fields.

**Fig 4:**
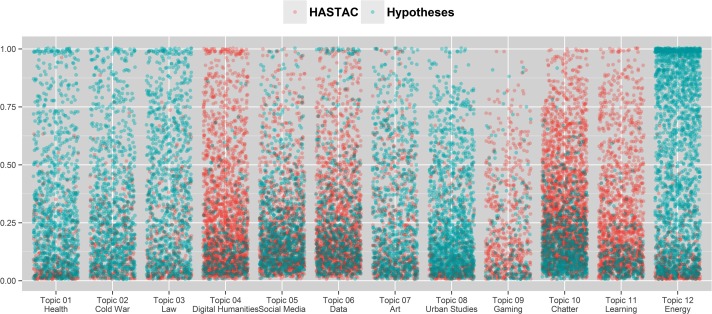
Distribution of posts per topic, with posts in red from HASTAC and posts in blue from Hypotheses.

The difference in the number of unique authors between the two platforms (923 authors on HASTAC vs. 403 authors on Hypotheses) may influence the result of the topic modeling, with a few very specific topics present on Hypotheses not represented on HASTAC (e.g. Cold War). Nonetheless, the results confirm the observations drawn from the co-word analysis, with topics on Hypotheses tending to be more disciplinarily aligned and connected exclusively to a single area of research, while posts on HASTAC are more likely to pick up interdisciplinary and general themes. Fig. 4 shows the topic scores in the 12 selected topics, with each dot representing a post and its color indicating the platform.

## Discussion

The results reported in this study can be summarized in two parts. Firstly, we found a substantial one-way dependency of DH terms on their unprefixed counterparts, as most blog posts dedicated to DH also included references to the corresponding humanities term (89% on HASTAC and 98% on Hypotheses). DH-related labels are considerably more frequent in HASTAC pointing to an unequal adoption of Digital Humanities-related terms in different local contexts. Secondly, we found a tendency in Hypotheses towards focused thematic areas representing disciplinary interests contrasted with a tendency to discuss more general, cross-disciplinary themes in HASTAC.

In terms of institutional branches of humanities research, history is the areas with the largest number of posts across the networks for the sample of topics considered in this study. Areas that are not traditionally associated with humanities research (or institutions that support the field), i.e. library and media, also account for a considerable portion of the posts. We also found considerable topical differences between the two platforms. While traditional areas of the humanities and social sciences (History, Art, Law) are clearly represented in Hypotheses, HASTAC is topically more cross-disciplinary and less focused on single disciplines. Some of these topics show considerable overlap between the networks (i.e. Social Media and Data), highlighting the fact that there are areas in which users of HASTAC and Hypotheses have similar interests, while others are considerably more predominant in one of the networks. Although both networks are on the forefront of the Digital Humanities research agenda, they present considerable differences in how explicitly they use new disciplinary labels (HASTAC) and address well-established disciplinary themes without explicitly associating them with DH (Hypotheses).

The differences we observed highlight that two platforms that attract broadly similar user communities may still differ considerably with regards to topics. We interpret the differences in adoption of Digital Humanities terminologies and topics across the networks to mirror different developments in DH. Whereas digital learning, digital literacy, and particularly digital scholarship are particularly prominent labels on HASTAC, Hypotheses is mostly focused on digital libraries, digital history, and digital archives. These differences are of qualitative and quantitative nature reflecting not just the personal preferences of bloggers and users, but may also indicate broader conceptual differences. While blog posts in HASTAC tend to raise issues suitable for (controversial) discussion, contributions in Hypotheses more closely mirror traditional expository humanities genres (e.g. book chapters or essays). Moreover, while HASTAC is a social network in which users can create profiles and interact with other users by posting and commenting on content, Hypotheses is a publishing platform with lesser emphasis on community building than HASTAC, and a closer alignment with traditional genres of publishing.

The content of each network also presents considerable variation in terms of formats and style. The prominence of Topic #12 (Chatter) in HASTAC indicates that HASTAC’s blog entries are conceptually more like casual conversation rather than academic writing. As blogs serve different purposes for different users, the data necessarily includes posts of different genres comprising of short essays, conference reviews, book reports, group discussions, and general academic advertising. While HASTAC and Hypotheses are interdisciplinary in character, they have a strong slant towards the humanities, particularly towards learning and digital media on HASTAC, and specifically towards history on Hypotheses. Common to both networks is the small proportion of users producing the large majority of the content, which leads to a typical long-tail distribution of content within the platforms.

In the last instance, the results reported in this study show that the variety of terms and topics associated with DH is locally configured and reflects different conceptualizations of what constitutes DH. We expect this study to be informative for future research grappling with the rapid establishment of DH in humanities departments. At any rate, it will be interesting to follow the ongoing maturation of both platforms and their respective approaches to scholarly blogging, as well as the different conceptualizations of Digital Humanities scholarship in North American and European contexts.

## Supporting Information

S1 Materials HASTAC dataset with 7,269 entries including timestamp and blog posts. (ZIP)

S2 Materials Hypotheses dataset with 6,777 entries including timestamp and blog posts. (ZIP)

## References

[1] Borgman CL (2007) Scholarship in the digital age: Information, infrastructure, and the Internet Cambridge, MA: MIT Press 360 p. 10.1093/jxb/erm028

[2] Meyer ET, Schroeder R (2009) The world wide web of research and access to knowledge. Knowl Manag Res Pract 7: 218–233. 10.1057/kmrp.2009.13.

[3] Nentwich M, König R (2012) Cyberscience 2.0: Research in the age of digital social networks Frankfurt am Main: Campus 237 p. 10.1007/s12070-012-0514-9

[4] Dutton WH, Jeffreys PW, editors (2010) World wide research: Reshaping the sciences and humanities. Cambridge, MA: MIT Press 408 p. 10.7551/mitpress/9780262014397.001.0001.

[5] Evans JA (2008) Electronic publication and the narrowing of science and scholarship. Science 321: 395–399. 10.1126/science.1150473 18635800

[6] Cope WW, Kalantzis M (2009) Signs of epistemic disruption: Transformations in the knowledge system of the academic journal. First Monday 14. Available: http://firstmonday.org/article/view/2309/2163. Accessed 23 December 2014.

[7] Mahrt M, Weller K, Peters I (2014) Twitter in scholarly communication In: Weller K, Bruns A, Burgess J, Mahrt M, Puschmann C, editors. Twitter and society. New York: Peter Lang pp. 399–410.

[8] Puschmann C, Mahrt M (2012) Scholarly blogging: A new form of publishing or science journalism 2.0? In: Tokar A, Beurskens M, Keuneke S, Mahrt M, Peters I, et al, editors. Science and the Internet. Düsseldorf: Düsseldorf University Press pp. 171–181.

[9] Puschmann C (2014) (Micro)blogging science? Notes on potentials and constraints of new forms of scholarly communication In: Bartling S, Friesike S, editors. Opening Science. Berlin, Heidelberg: Springer International Publishing pp. 89–106. 10.1007/978-3-319-00026-8.

[10] Shema H, Bar-Ilan J, Thelwall M (2012) Research blogs and the discussion of scholarly information. PLoS One 7: e35869 10.1371/journal.pone.0035869 22606239 PMC3350512

[11] Kjellberg S (2010) I am a blogging researcher: Motivations for blogging in a scholarly context. First Monday 15. Available: http://firstmonday.org/article/view/2962/2580. Accessed 23 December 2014.

[12] Gruzd A, Staves K, Wilk A (2012) Connected scholars: Examining the role of social media in research practices of faculty using the UTAUT model. Comput Human Behav 28: 2340–2350. 10.1016/j.chb.2012.07.004.

[13] Rowlands I, Nicholas D, Russell B, Canty N, Watkinson A (2011) Social media use in the research workflow. Learn Publ 24: 183–195. 10.1087/20110306.

[14] Priem J, Hemminger BH (2010) Scientometrics 2.0: New metrics of scholarly impact on the social web. First Monday 15. Available: http://firstmonday.org/article/view/2874/2570. Accessed 23 December 2014.

[15] Bar-Ilan J, Haustein S, Peters I, Priem J, Shema H, et al (2012) Beyond citations: Scholars’ visibility on the social web In: Archambault É, Gingras Y, Larivière V, editors. Proceedings of the 17th International Conference on Science and Technology Indicators. Montréal: Science-Metrix and OST pp. 98–109.

[16] Schreibman S, Siemens R, Unsworth JM, editors (2004) A companion to digital humanities Oxford: Blackwell Publishers 604 p.

[17] Gold MK, editor (2012) Debates in the digital humanities Minneapolis, MN: University of Minnesota Press 532 p. 10.1007/s12070-012-0514-9

[18] Berry D (2012) Understanding digital humanities Basingstoke: Palgrave Macmillan 336 p. 10.1007/s12070-012-0514-9

[19] Kirschenbaum MG (2010) What is digital humanities and what’s it doing in English departments? ADE Bull 150: 55–61.

[20] Juola P (2008) Killer applications in digital humanities. Lit Linguist Comput 23: 73–83. 10.1093/llc/fqm042.

[21] Moretti F (2007) Graphs, maps, trees: Abstract models for a literary history New York: Verso 119 p. 10.1016/j.encep.2007.07.008

[22] McPherson T (2008) Introduction: Media studies and the digital humanities. Cine J 48: 119–123. 10.1353/cj.0.0077.

[23] Pinch TJ, Bijker WE (1984) The social construction of facts and artefacts: Or how the sociology of science and the sociology of technology might benefit each other. Soc Stud Sci 14: 399–441. 10.1177/030631284014003004.

[24] Ross C, Terras M, Warwick C, Welsh A (2011) Enabled backchannel: Conference Twitter use by digital humanists. J Doc 67: 214–237. 10.1108/00220411111109449.

[25] Puschmann C, Weller K, Dröge E (2011) Studying Twitter conversations as (dynamic) graphs: Visualization and structural comparison In: Taddicken M, editor. Proceedings of General Online Research 2011. Düsseldorf: DGOF.

[26] Yan E, Ding Y (2012) A framework of studying scholarly networks. In: Archambault É, Gingras Y, Larivière V, editors. Proceedings of the 17th International Conference on Science and Technology Indicators. Montréal: Science-Metrix and OST. pp. 917–926.

[27] Davidson CN, Goldberg DT (2004) A manifesto for the humanities in a technological age. Chron High Educ: B7.

[28] HASTAC (2014) About HASTAC. Available: http://www.hastac.org/about. Accessed 23 December 2014.

[29] Hypotheses.org (2014) About Hypotheses. Available: http://hypotheses.org/about/hypotheses-org-en. Accessed 23 December 2014.

[30] Lui M, Baldwin T (2012) langid.py: An off-the-shelf language identification tool. In: Li H, editor. Proceedings of the 50th Annual Meeting of the Association for Computational Linguistics. Jeju Island, Korea: ACL. pp. 25–30.

[31] Callon M, Courtial J-P, Turner WA, Bauin S (1983) From translations to problematic networks: An introduction to co-word analysis. Soc Sci Inf 22: 191–235. 10.1177/053901883022002003.

[32] Taddy M (2013) Multinomial inverse regression for text analysis. J Am Stat Assoc 108: 755–770. 10.1080/01621459.2012.734168.

[33] Lipsitz SR, Laird NM, Harrington DP (1991) Generalized estimating equations for correlated binary data: Using the odds ratio as a measure of association. Biometrika 78: 153–160. 10.1093/biomet/78.1.153.

[34] Moody J, Light R (2006) A view from above: The evolving sociological landscape. Am Sociol 37: 67–86. 10.1007/s12108-006-1006-8.

[35] Leydesdorff L (2004) Top-down decomposition of the Journal Citation Report of the Social Science Citation Index: Graph- and factor-analytical approaches. Scientometrics 60: 159–180. 10.1023/B:SCIE.0000027678.31097.e0.

[36] Kruskal JB (1964) Multidimensional scaling by optimizing goodness of fit to a nonmetric hypothesis. Psychometrika 29: 1–27. 10.1007/BF02289565.

[37] Moody J (2004) The structure of a social science collaboration network: Disciplinary cohesion from 1963 to 1999. Am Sociol Rev 69: 213–238. 10.1177/000312240406900204.

[38] Wickham H (2009) ggplot2: Elegant graphics for data analysis Berlin, Heidelberg: Springer 213 p.

[39] Blei DM, Ng AY, Jordan MI (2003) Latent dirichlet allocation. J Mach Learn Res 3: 993–1022. 10.1162/jmlr.2003.3.4-5.993.

[40] Grün B, Hornik K (2011) topicmodels: An R package for fitting topic models. J Stat Softw 40 22523482

[41] Feinerer I, Hornik K, Meyer D (2008) Text mining infrastructure in R. J Stat Softw 25.

